# Surgical and multimodal treatment of metastatic oesophageal cancer: retrospective cohort study

**DOI:** 10.1093/bjsopen/zrae054

**Published:** 2024-05-30

**Authors:** Karl Knipper, Julian Lemties, Thaddaeus Krey, Su Ir Lyu, Naita M Wirsik, Lars M Schiffmann, Hans F Fuchs, Florian Gebauer, Wolfgang Schröder, Felix C Popp, Alexander Quaas, Hans A Schlößer, Christiane J Bruns, Thomas Schmidt

**Affiliations:** Department of General, Visceral and Cancer Surgery, Faculty of Medicine and University Hospital of Cologne, University of Cologne, Cologne, Germany; Department of General, Visceral and Cancer Surgery, Faculty of Medicine and University Hospital of Cologne, University of Cologne, Cologne, Germany; Department of General, Visceral and Cancer Surgery, Faculty of Medicine and University Hospital of Cologne, University of Cologne, Cologne, Germany; Institute of Pathology, Faculty of Medicine and University Hospital of Cologne, University of Cologne, Cologne, Germany; Department of General, Visceral and Cancer Surgery, Faculty of Medicine and University Hospital of Cologne, University of Cologne, Cologne, Germany; Department of General, Visceral and Cancer Surgery, Faculty of Medicine and University Hospital of Cologne, University of Cologne, Cologne, Germany; Department of General, Visceral and Cancer Surgery, Faculty of Medicine and University Hospital of Cologne, University of Cologne, Cologne, Germany; Department of General, Visceral and Cancer Surgery, Faculty of Medicine and University Hospital of Cologne, University of Cologne, Cologne, Germany; Department of General, Visceral and Cancer Surgery, Faculty of Medicine and University Hospital of Cologne, University of Cologne, Cologne, Germany; Department of General, Visceral and Cancer Surgery, Faculty of Medicine and University Hospital of Cologne, University of Cologne, Cologne, Germany; Institute of Pathology, Faculty of Medicine and University Hospital of Cologne, University of Cologne, Cologne, Germany; Department of General, Visceral and Cancer Surgery, Faculty of Medicine and University Hospital of Cologne, University of Cologne, Cologne, Germany; Center for Molecular Medicine Cologne, Faculty of Medicine and University Hospital of Cologne, University of Cologne, Cologne, Germany; Department of General, Visceral and Cancer Surgery, Faculty of Medicine and University Hospital of Cologne, University of Cologne, Cologne, Germany; Department of General, Visceral and Cancer Surgery, Faculty of Medicine and University Hospital of Cologne, University of Cologne, Cologne, Germany

## Abstract

**Background:**

In contrast to the well-established multimodal therapy for localized oesophageal cancer, the metastatic stage is commonly treated only with systemic therapy as current international guidelines recommend. However, evidence suggesting that multimodal therapy including surgery could benefit selected patients with metastasized oesophageal cancer is increasing. The aim of this study was to investigate the survival of patients diagnosed with metastatic oesophageal cancer after different treatment regimens.

**Methods:**

This was a retrospective single-centre study of patients with adenocarcinoma or squamous cell carcinoma of the oesophagus with synchronous or metachronous metastases who underwent Ivor Lewis oesophagectomy between 2010 and 2021. Each patient received an individual treatment for their metastatic burden based on an interdisciplinary tumour board conference. Survival differences between different treatments were assessed using the Kaplan–Meier method, as well as univariable and multivariable Cox regression models.

**Results:**

Out of 1791 patients undergoing Ivor Lewis oesophagectomy, 235 patients diagnosed with metastases were included. Of all of the included patients, 42 (17.9%) only underwent surgical resection of their metastatic disease, 37 (15.7%) underwent multimodal therapy including surgery, 78 (33.2%) received chemotherapy alone, 49 (20.9%) received other therapies, and 29 (12.3%) received best supportive care. Patients who underwent resection or multimodal therapy including surgery of their metastatic burden showed superior overall survival compared with chemotherapy alone (median overall survival of 19.0, 18.0, and 11.0 months respectively) (*P* < 0.001). This was confirmed in subcohorts of patients with metachronous solid-organ metastases and with a single metastasis. In multivariable analyses, resection with or without multimodal therapy was an independent factor for favourable survival.

**Conclusion:**

Surgical resection could be a feasible treatment option for metastasized oesophageal cancer, improving survival in selected patients. Further prospective randomized studies are needed to confirm these findings and define reliable selection criteria.

## Teaser

This retrospective single-centre cohort study included 235 patients with metastasized oesophageal cancer. A comparison was conducted of patient outcomes after resection alone, multimodal therapy including surgery, chemotherapy alone, any other therapy without surgery, or best supportive care as a treatment for metastasized oesophageal cancer. Resection or multimodal therapy including surgery resulted in the best overall survival in the total cohort and in subcohorts of patients with metachronous solid-organ metastases, with metastases in the liver, lung, or adrenal gland, or with a single metastasis.

## Introduction

Oesophageal cancer is one of the most malignant cancer entities, with a worldwide incidence that is increasing. Cancer deaths are estimated to reach nearly 1 million patients per year in 2040^1^. Treatment options are limited, especially for patients diagnosed with distant metastases. The median overall survival from the time of treatment assignment until death in controlled trials is around 11 months^2^. Previous retrospective studies described the metastatic patterns of oesophageal cancer. The liver is the most affected organ, followed by the lung, distant lymph nodes, bones, and the brain^3,4^. Multimodality therapies including surgery and perioperative chemotherapy/chemoradiotherapy/radiotherapy have been implemented as the standard of care for locally advanced oesophageal cancer^5,6^. In contrast, current guidelines recommend treating patients with unresectable or metastatic oesophageal cancer only with immune checkpoint inhibitors and/or chemotherapy^7^. Data demonstrating the benefit of surgery for metastatic oesophageal cancer are still limited and many centres do not consider surgery for oligometastatic disease^8^, which is currently considered to be incurable^7^. In contrast, local therapy of metastases is recommended and accepted for other tumour entities, such as colorectal cancer^9^. In a retrospective cohort study of patients with metastasized colorectal carcinoma, for example, liver resection proved to be a factor for favourable survival^10^. Recently, more data on patients undergoing resection of metastases of cancer entities of the upper gastrointestinal tract were published. In the German AIO-FLOT3 trial, 36 patients with limited metastasized gastric or gastro-oesophageal junction cancer showed a median overall survival of 31.3 months after multimodal therapy including resection of the primary tumour and metastases^11^. Patients who did not undergo resection showed a median overall survival of 15.9 months^11^. These data demonstrate that selected patients with stage IV cancer of the upper gastrointestinal tract show favourable long-term survival after surgical resection. Multimodality treatment including surgery has therefore been considered for selected patients with limited metastatic disease. The aim of this study was to investigate the hypothesis that multimodality therapy including resection is superior to chemotherapy alone for patients after oesophagectomy with limited metastatic disease.

## Methods

### Patient cohort

Patients with synchronous or metachronous metastases of oesophageal cancer were selected from a prospective database of patients undergoing Ivor Lewis oesophagectomy with curative intent between 2010 and 2021. Written informed consent for inclusion in the prospective database was obtained from every enrolled patient. The present retrospective analysis was approved by the local ethics committee (reference no. 13-091). The study was conducted in accordance with the Declaration of Helsinki and is reported in line with the STROCSS (Strengthening The Reporting Of Cohort Studies in Surgery) criteria^12^. This trial was not pre-registered in an independent institutional registry.

Follow-up imaging was performed at least yearly. Metastatic disease was diagnosed by imaging (CT or MRI) or by histology (after operative resection or biopsy). Upon diagnosis of metastases, all patients were discussed in an interdisciplinary tumour board conference and treated based on individual tumour board concepts. Criteria for eligibility for resection were the possibility of complete resection, limited metastatic burden, and the patient’s general condition. Operative resection was conducted at the University Hospital of Cologne.

Subgroup analyses were conducted for patients with metachronous solid-organ metastases, with metastases in the liver, lung, or adrenal gland, or with a single metastasis. Additionally, an independent treatment-naive patient cohort, consisting of patients diagnosed with distant lymph node metastases (cM1) using PET between 2014 and 2022, was included. Resection of the primary tumour was performed with curative intent.

Variables analysed were overall survival, age, sex, ASA grade, Siewert classification of the adenocarcinoma of the oesophagogastric junction, histology, neoadjuvant treatment of the primary tumour, TNM stage, time between primary surgery and occurrence of metastasis, number of metastases, number of metastatic sites, and treatment of metastatic disease.

#### Definition of individualized treatment strategies in limited metastatic disease

Limited metastatic disease was defined as up to two metastases at the time of diagnosis of the metastatic burden. Pleural, peritoneal, and meningeal carcinomatosis were excluded from graded limited metastatic disease. Local therapy (resection or radiotherapy) of the mentioned limited metastatic disease was defined as individualized therapy. In contrast, all other therapy regimens were defined as palliative treatment. Patients who did not receive any treatment were described as having received ‘best supportive care’.

### Statistical analysis

Overall survival was defined as the time between operative resection of the metastasis, the beginning of chemotherapy/chemoradiotherapy/radiotherapy, or the date of diagnosis of the metastasis when no further therapy was performed and patient death or loss to follow-up. Reverse Kaplan–Meier was used to calculate the median overall survival. Survival analyses were performed using the Kaplan–Meier method and the log rank test was used to test for differences between groups. Interdependencies between survival and clinicopathological values were calculated using univariable and multivariable Cox regression. Variables with *P* < 0.200 in the univariable Cox regression analyses were included in the multivariable Cox regression analyses. Collinearity was considered by calculating the variance inflation factor before multivariable Cox regression. Parameters with values greater than three were excluded. Qualitative values are reported as percentages and were compared using the chi-squared test and numerical values are reported as means and were compared using Student’s *t* test. A two-tailed *P* < 0.050 was considered statistically significant. All analyses were performed using SPSS^®^ (IBM, Armonk, NY, USA; Statistics, version 28.0.1.1).

## Results

### Characteristics of the primary tumour

**Fig. 1:**
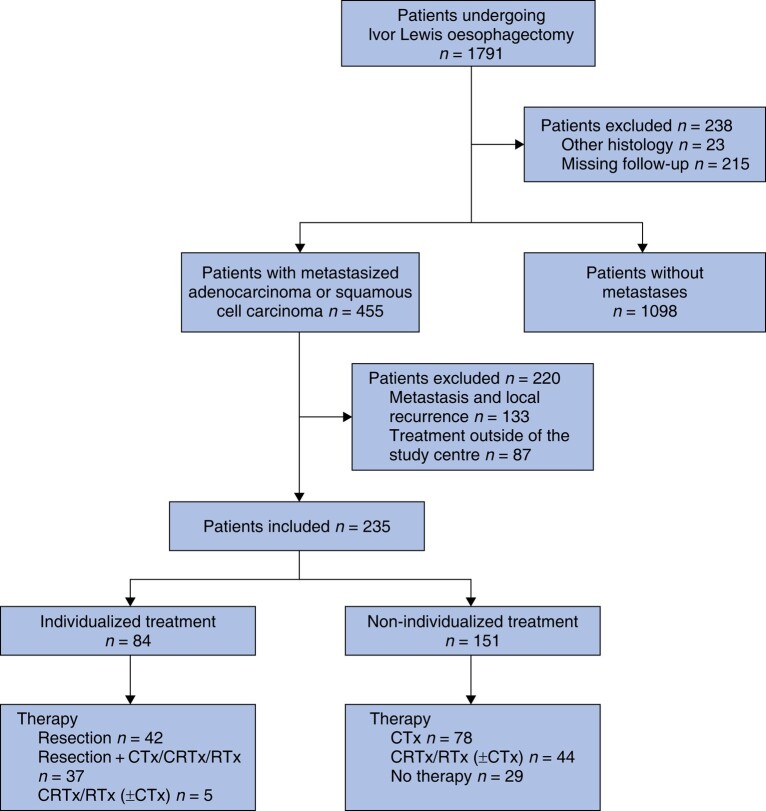
Flow chart of the inclusion process CTx, chemotherapy; CRTx, chemoradiotherapy; RTx, radiotherapy.

A total of 1791 patients, who underwent Ivor Lewis oesophagectomy during the study interval, were screened (*Fig. 1*). The following patients were excluded: patients not diagnosed with metastasis during follow-up (1098 patients, 61.3%); patients with tumour entities other than adenocarcinoma or squamous cell carcinoma (23 patients); patients with missing follow-up (215 patients); and patients diagnosed with combined local recurrence and metastasis (133 patients). Furthermore, only patients who were treated for their metastatic oesophageal cancer at the University Hospital of Cologne were included in the present study.

Finally, 235 patients (13.1%) diagnosed with metastatic oesophageal cancer synchronous or metachronous to Ivor Lewis oesophagectomy were analysed. Of the total cohort, 84.3% were males. On the date of resection of the primary tumour, 154 patients (65.5%) were less than 65 years old. The majority of the included patients (83.8%) were diagnosed with adenocarcinoma of the oesophagus; 16.2% were diagnosed with squamous cell carcinoma. Most patients (85.5%) received neoadjuvant therapy before the resection of the primary tumour and the T3 stage was the predominant pathological T stage. In 60.0% of the patients, pathological lymph node metastases were diagnosed. In 93.6% of the patients a complete resection of the primary tumour was accomplished. General patient characteristics are given in *Table 1* and a detailed listing of postoperative complications after Ivor Lewis oesophagectomy is given in *Table S1*.

**Table 1:** General patient characteristics and clinicopathological characteristics regarding the primary tumour of the whole study population, as well as the patient cohorts receiving individualized treatment and non-individualized treatment

Characteristic	Total		Individualized treatment		Non-individualized treatment		*P*
Patients	235 (100.0)		84 (100.0)		151 (100.0)		
**Sex**							0.074
Male	198 (84.3)		66 (78.6)		132 (87.4)		
Female	37 (15.7)		18 (21.4)		19 (12.6)		
**Age (years)**							0.156
<65	154 (65.5)		60 (71.4)		94 (62.3)		
65	81 (34.5)		24 (28.6)		57 (37.7)		
**ASA grade**							0.786
I	30 (12.8)		12 (14.3)		18 (11.9)		
II	106 (45.1)		40 (47.6)		66 (43.7)		
III	60 (25.5)		20 (23.8)		40 (26.5)		
IV	0 (0.0)		0 (0.0)		0 (0.0)		
Unknown	39 (16.6)		12 (14.3)		27 (17.9)		
**Histology**							0.372
Adenocarcinoma	197 (83.8)		68 (81.0)		129 (85.4)		
Squamous cell carcinoma	38 (16.2)		16 (19.0)		22 (14.6)		
**Siewert classification of the adenocarcinoma of the oesophagogastric junction**							0.887
1	78 (33.2)		26 (30.1)		52 (34.3)		
2	70 (29.8)		26 (30.1)		44 (29.1)		
3	3 (1.3)		1 (1.2)		2 (1.3)		
Unknown/not applicable	84 (35.7)		31 (36.9)		53 (35.1)		
**Neoadjuvant therapy**							0.147
No	23 (9.8)		5 (6.0)		18 (11.9)		
Yes	201 (85.5)		75 (89.3)		126 (83.4)		
Unknown	11 (4.7)		4 (4.7)		7 (4.7)		
	**Neoadjuvant**	**Non-neoadjuvant**	**Neoadjuvant**	**Non-neoadjuvant**	**Neoadjuvant**	**Non-neoadjuvant**	
**pT**							0.007*/0.026*/0.194
0	30 (14.9)	0 (0.0)	16 (21.3)	0 (0.0)	14 (11.1)	0 (0.0)	
1	32 (15.9)	2 (5.9)	11 (14.7)	0 (0.0)	21 (16.7)	2 (8.0)	
2	34 (16.9)	10 (29.4)	18 (24.0)	5 (55.6)	16 (12.7)	5 (20.0)	
3	98 (48.8)	20 (58.8)	27 (36.0)	4 (44.4)	71 (56.3)	16 (64.0)	
4	7 (3.5)	2 (5.9)	3 (4.0)	0 (0.0)	4 (3.2)	2 (8.0)	
**pN**							0.224/0.061/0.485
0	84 (41.8)	10 (29.4)	38 (50.7)	2 (22.2)	46 (36.5)	8 (32.0)	
1	56 (27.9)	8 (23.5)	22 (29.3)	1 (11.1)	34 (27.0)	7 (28.0)	
2	38 (18.9)	4 (11.8)	11 (14.7)	1 (11.1)	27 (21.4)	3 (12.0)	
3	23 (1.4)	12 (35.3)	4 (5.3)	5 (55.6)	19 (15.1)	7 (28.0)	
**R**							0.145/0.224/0.454
0	193 (96.0)	27 (79.4)	73 (97.3)	6 (66.7)	120 (95.2)	21 (84.0)	
1	4 (2.0)	2 (5.9)	0 (0.0)	0 (0.0)	4 (3.2)	2 (8.0)	
2	1 (0.5)	0 (0.0)	0 (0.0)	0 (0.0)	1 (0.8)	0 (0.0)	
Unknown	3 (1.5)	5 (14.7)	2 (2.7)	3 (33.3)	1 (0.8)	3 (12.0)	
**Lymphatic vessel invasion**							0.204/0.170/0.430
0	126 (62.7)	12 (35.3)	53 (70.7)	2 (22.2)	73 (57.9)	10 (40.0)	
1	63 (31.3)	17 (50.0)	20 (26.7)	5 (55.6)	43 (34.1)	12 (48.0)	
Unknown	12 (6.0)	5 (14.7)	2 (2.7)	2 (22.2)	10 (8.0)	3 (12.0)	
**Blood vessel invasion**							0.112/0.271/0.129
0	162 (80.6)	24 (70.6)	64 (85.3)	7 (77.8)	98 (77.8)	16 (64.0)	
1	28 (13.9)	5 (14.7)	8 (10.7)	0 (0.0)	20 (15.9)	5 (20.0)		
Unknown	11 (5.5)	5 (14.7)	3 (4.0)	2 (22.2)	8 (6.3)	4 (16.0)	

Values are *n* (%). TNM status is reported for neoadjuvant-treated and non-neoadjuvant-treated (primary operation and unknown neoadjuvant status) patients separately. *P* values are for comparisons of the whole cohort/only neoadjuvant-treated patients/only non-neoadjuvant-treated patients. *Statistically significant.

### Site and treatment of the metastatic disease

Of the included patients, 83.8% had metachronous metastases (*Table 2*) and 138 (70.1%) developed metastasis during the first 12 months after resection of the primary tumour. At the initial diagnosis of the metastatic disease, 22.6% of all patients were diagnosed with more than one metastatic lesion. The most common metastatic site was the liver (58 patients, 24.7%), followed by the lung (43 patients, 18.3%), distant lymph nodes (25 patients, 10.6%), and the peritoneum (25 patients, 10.6%) (*Table S2*). A total of 37 patients (15.7%) received multimodal therapy including resection of the metastatic burden. Resection without further oncological treatment was performed in 42 patients (17.9%); additional patients received chemoradiotherapy or radiotherapy (±chemotherapy) (20.9%), chemotherapy alone (33.2%), or best supportive care (12.3%). Overall, 35.7% of all of the included patients were treated with an individualized treatment plan with curative intent. The median follow-up was 20 months and 1-, 2-, and 3-year overall survival rates were 44.3%, 23.6%, and 15.6% respectively.

**Table 2:** Patient characteristics regarding the metastatic disease of the whole study population, as well as the patient cohorts receiving individualized treatment and non-individualized treatment

Characteristic	Total	Individualized treatment	Non- individualized treatment	*P*
**Patients**	235 (100.0)	84 (100.0)	151 (100.0)	
**Metachronous metastases**				<0.001*
Yes	197 (83.8)	60 (71.4)	137 (90.7)	
No	38 (16.2)	24 (28.6)	14 (9.3)	
**Time of metachronous metastases after primary surgery (months)**				0.042*
<13	138 (70.1)	36 (60.0)	102 (74.5)	
≥13	59 (29.9)	24 (40.0)	35 (25.5)	
**Number of metastases at first diagnosis of metastases**				<0.001*
1	182 (77.4)	78 (92.9)	104 (68.9)	
2	43 (18.3)	6 (7.1)	37 (24.5)	
3	7 (3.0)	0 (0.0)	7 (4.6)	
≥4	3 (1.3)	0 (0.0)	3 (2.0)	
**Number of metastatic sites at first diagnosis of metastases**				<0.001*
1	186 (79.1)	80 (95.2)	106 (70.2)	
2	33 (14.0)	4 (4.8)	29 (19.2)	
≥3	16 (6.8)	0 (0.0)	16 (10.6)	
**Intention of treatment**				–
Palliative	122 (51.9)	0 (0.0)	122 (80.8)	
Individualized	84 (35.7)	84 (100.0)	0 (0.0)	
Best supportive care	29 (12.3)	0 (0.0)	29 (19.2)	
**Resection of metastases**				–
No	156 (66.4)	5 (6.0)	122 (100.0)	
Yes	79 (33.6)	79 (94.0)	0 (0.0)	
**Treatment**				–
Resection	42 (17.9)	42 (50.0)	0 (0.0)	
Resection + CTx/CRTx/RTx	37 (15.7)	37 (44.0)	0 (0.0)	
CTx	78 (33.2)	0 (0.0)	78 (51.7)	
CRTx/RTx (±CTx)	49 (20.9)	5 (6.0)	44 (29.1)	
No therapy	29 (12.3)	0 (0.0)	29 (19.2)	

Values are *n* (%). *Statistically significant. CTx, chemotherapy; CRTx, chemoradiotherapy; RTx, radiotherapy.

### Individualized treatment *versus* non-individualized treatment

Patients in the individualized treatment group had significantly lower pT stages (*P* < 0.001) (*Table 1*) with regard to the primary tumour. No other significant differences were found regarding the characteristics of the primary tumour. Local treatment was more often performed for patients with metachronous metastases (*P* < 0.001) (*Table 2*). The majority of the included patients (94.0%) in the individualized treatment group underwent complete resection of their metastatic burden. Unimodal resection was performed for 42 patients (50.0%) in the individualized treatment group, 37 patients (44.0%) underwent resection and also received chemotherapy, chemoradiotherapy, or radiotherapy, and 5 patients (6.0%) received chemoradiotherapy or radiotherapy (±chemotherapy). A detailed listing of the administered systemic therapy regimens is given in *Table S3*. Patients with synchronous metastasis mainly received FLOT (Fluorouracil, Leucovorin, Oxaliplatin and Docetaxel) as perioperative treatment according to the FLOT-3 trial. In contrast, patients who developed metastasis metachronously received a variety of treatment regimens, including immunotherapy. Comparing postoperative complications between patients with synchronous metastatic disease with simultaneous metastases resection and patients with synchronous metastatic disease without simultaneous metastases resection showed no significant difference regarding frequent and severe postoperative complications, such as the development of anastomotic leakage (20.8% *versus* 18.2% respectively) (*P* = 0.856) (*Table S1*).

### Survival analyses of the total patient cohort

**Fig. 2:**
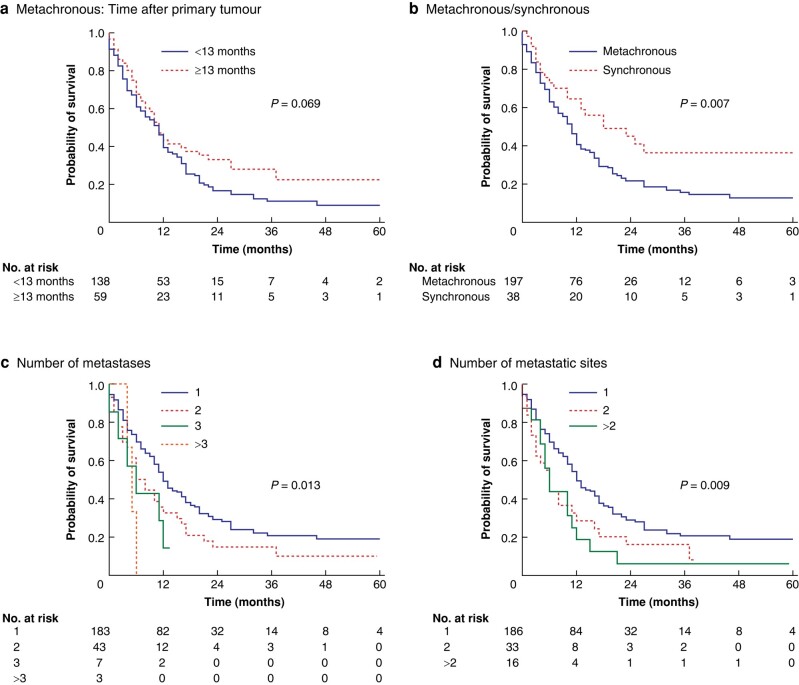

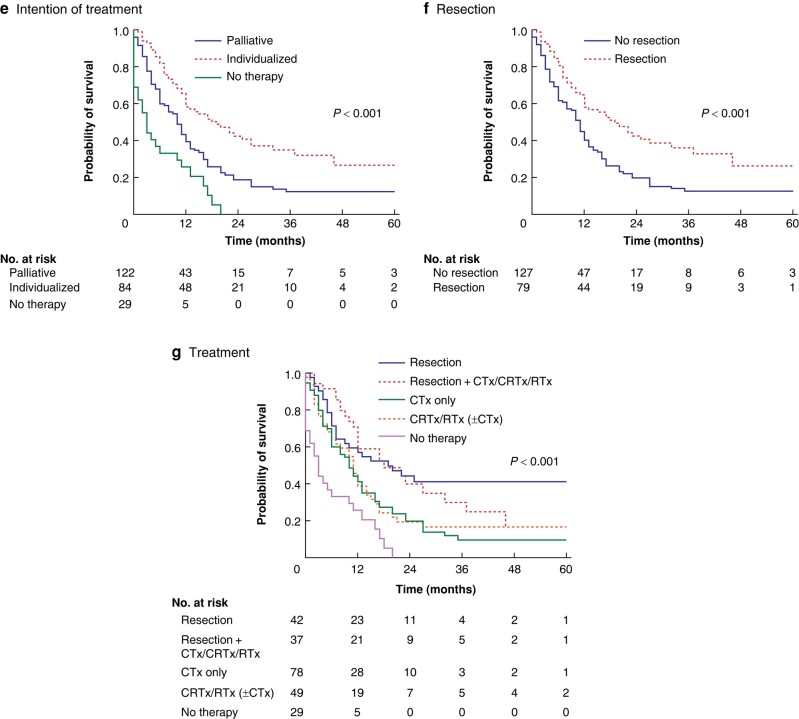
Kaplan–Meier curves for overall survival depending on various factors **a** Time of diagnosis of the metastatic disease after primary surgery. **b** Metachronous or synchronous metastatic disease. **c** Number of metastases. **d** Number of metastastic sites. **e** Intention of treatment. **f** Surgical resection. **g** Treatment option. CTx, chemotherapy; CRTx, chemoradiotherapy; RTx, radiotherapy.

To evaluate the impact of the duration between surgery and the onset of metastatic disease, patients were categorized into early relapse (less than 13 months) and late relapse (greater than or equal to 13 months). The time of diagnosis had no influence on survival in this patient cohort (*Fig. 2a*). However, patients with metachronous metastatic disease showed significantly worse overall survival compared with patients with synchronous metastases (*P* = 0.007) (*Fig. 2b*). A higher number of metastases (*P* = 0.013) (*Fig. 2c*) and a higher number of metastatic sites (*P* = 0.009) (*Fig. 2d*) also correlated with worse overall survival. Patients who received individualized treatment for their metastatic disease showed significantly longer survival compared with the patients who received palliative treatment or best supportive care (*P* < 0.001) (*Fig. 2e*). Next, patients receiving any treatment for their metastatic burden were separated into a resected group and a non-resected group; the resected group showed a significantly prolonged median survival (19.0 (95% c.i. 11.85 to 26.15) months) compared with the non-resected group (11.0 (95% c.i. 9.44 to 12.56) months) (*P* < 0.001) (*Fig. 2f*). These findings were confirmed for the detailed therapy regimens, with resection resulting in the best survival compared with all the other treatments (*P* < 0.001) (*Fig. 2g*). Furthermore, univariable Cox regression analyses were conducted to further investigate factors that could influence patient survival (*Table S4*). Known risk factors, such as pN, pT, and R status, lymphatic vessel invasion of the primary tumour, and a higher number of metastases or metastatic sites, proved to be factors for worse patient survival. Neoadjuvant therapy of the primary tumour, synchronous metastatic disease, and resection of the metastatic disease were predictive factors for increased patient survival. After adjustment for collinearity, multivariable Cox regression analyses were performed to correct for potential confounders. Time of onset of metachronous disease and number of metastatic sites were excluded from the multivariable Cox regression analysis due to collinearity, whereas patient age, neoadjuvant therapy, pT, pN, and R status, lymphatic and blood vessel invasion, onset of metastatic disease, number of metastases, and treatment of the metastatic burden were included. In multivariable analysis, a higher pN status was an independent risk factor for inferior overall survival in patients with metastases. Furthermore, resection of the metastatic burden proved to be an independent factor for favourable survival (*Table 3*).

**Table 3:** Multivariable cox regression

Characteristic	Comparison groups	HR (95% c.i.)	*P*
Age (years)	≥65 *versus* <65	1.03 (0.68, 1.56)	0.885
Neoadjuvant therapy	yes *versus* no	0.79 (0.40, 1.59)	0.514
pT	≥2 *versus* 1	0.86 (0.71, 1.05)	0.141
pN	≥1 *versus* 0	1.29 (1.05, 1.60)	0.017*
R	≥1 *versus* 0	3.86 (0.99, 15.15)	0.053
Lymphatic vessel invasion	1 *versus* 0	1.13 (0.72, 1.80)	0.594
Blood vessel invasion	1 *versus* 0	1.20 (0.69, 2.10)	0.523
Metachronous metastases	no *versus* yes	0.65 (0.36, 1.18)	0.153
Number of metastases	≥2 *versus* 1	1.35 (0.96, 1.88)	0.085
Resection	yes *versus* no	0.64 (0.42, 0.97)	0.034*

^*^Statistically significant.

### Tumour histology and onset of metastases

To further determine patient groups that could profit from individualized treatment, survival analyses were conducted for different subcohorts. First, the total cohort was divided into those with adenocarcinoma and those with squamous cell carcinoma. Individualized treatment resulted in better survival in patients with adenocarcinoma compared with palliative treatment and best supportive care (*P* < 0.001) (*Fig. S1a*); however, resection did not reach statistical significance in multivariable Cox regression analyses (HR 0.65, 95% c.i. 0.41 to 1.03; *P* = 0.064). For patients with squamous cell carcinoma, no differences were found between the different treatment regimens (*P* = 0.221) (*Fig. S1b*). Furthermore, the onset of metastases was investigated. Patients with metachronous disease could benefit from individualized treatment (*P* < 0.001) (*Fig. S1c*). Moreover, patients with the onset of metastatic disease both within 1 year and greater than 1 year after primary tumour resection showed improved survival after individualized treatment (*Fig. S1d*,*e*). No difference in overall survival after different treatment options was observed for patients with synchronous metastatic disease (*Fig. S1f*).

### Metachronous solid-organ metastases

**Fig. 3:**
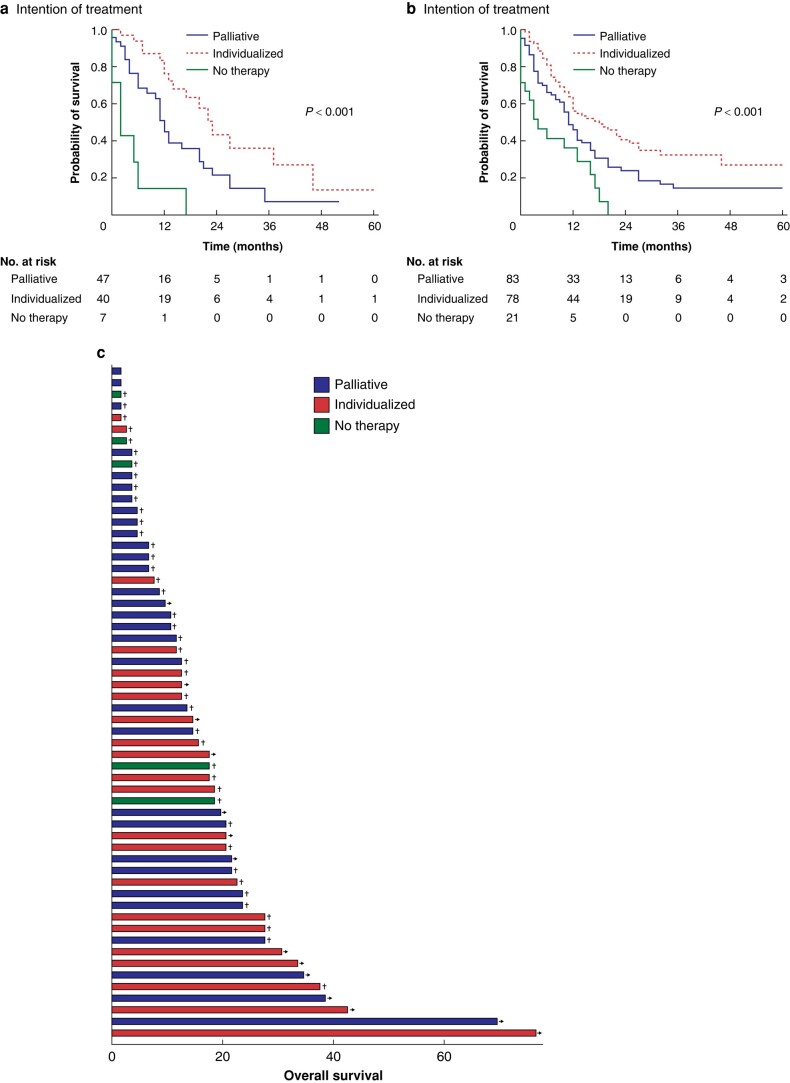
Kaplan–Meier curves for overall survival depending on the intention of treatment **a** For patients with metachronous solid-organ metastases. **b** For patients with a single metastasis. **c** Exemplary swimmer plot for overall survival in months of patients with liver metastasis depending on the intention of treatment (palliative, *n* = 31, individualized, *n* = 22; and no therapy, *n* = 5) (*P* = 0.016). →: patient alive; †: patient dead.

Further subgroup analyses were performed for 94 patients diagnosed with metachronous solid-organ metastases (liver, 44 patients; lung, 34 patients; or adrenal gland, 16 patients) after resection of the primary tumour. Patients who received individualized therapy showed a significantly better median overall survival (individualized: 23.0 (95% c.i. 5.56 to 40.44) months; palliative: 12.0 (95% c.i. 9.90 to 14.10) months; and no therapy: 2.0 (95% c.i. 0.00 to 4.57) months) (*P* < 0.001) (*Fig. 3a*).

### Metastases in the liver, lung, or adrenal gland, or a single metastasis

To evaluate the different therapy strategies depending on the site of metastasis, further sub-analyses were conducted for each metastatic site. Individualized therapy was shown to be a superior treatment option for patients with a single metastasis (*P* < 0.001) (*Fig. 3b*). Additionally, individualized therapy was associated with the best overall survival whether the site of metastasis was in the liver, lung, or adrenal gland (*Fig. 3c* and *Fig. S2a–c*).

### Patients with preoperative PET-positive distant lymph node metastases

Lastly, a separate cohort of 17 patients diagnosed before surgery with distant lymph node metastases using PET was investigated (*Table S5*). The majority of these patients underwent neoadjuvant therapy (82.4%), followed by Ivor Lewis oesophagectomy. Additionally, the cM1+ lymph nodes were treated: seven patients (41.2%) received adjuvant radiotherapy for the PET-positive lymph node area, six patients (35.3%) underwent extended lymphadenectomy, two patients (11.8%) received adjuvant chemotherapy, one patient (5.9%) received adjuvant chemoradiotherapy, and one patient (5.9%) underwent active surveillance regarding the PET-positive lymph nodes. The specimens after Ivor Lewis oesophagectomy were analysed for pathological lymph node metastases. No pathological lymph nodes were detected in 6 of 17 (35.3%) patients with PET-positive distant lymph node metastases. Pathological lymph node metastases were diagnosed in 11 patients (64.7%). Survival analyses showed that, regardless of their initial PET-positive lymph nodes, patients without histological evidence of lymph node metastases showed significantly better survival compared with patients with lymph node metastases (median overall survival: pN0, not reached; and pN+, 18.0 (95% c.i. 15.0 to 21.0) months) (*P* = 0.011) (*Fig. S3a*,*b*).

## Discussion

This retrospective analysis of 235 patients with oesophageal adenocarcinoma or squamous cell carcinoma after Ivor Lewis oesophagectomy and diagnosed with metastatic disease demonstrated that patients who underwent surgical resection of their metastases, including patients who received multimodal therapy, showed significantly better overall survival compared with patients who received other treatment regimens. This was confirmed in subgroups of patients diagnosed with metachronous solid-organ metastases, with metastases in the liver, lung, or adrenal gland, or with a single metastasis.

In recent years, there is increasing evidence that local therapy for metastasized oesophageal cancer, such as resection or radiation, is a feasible treatment option for selected patients^13–15^. However, the comparison of patients who received multimodal therapy including resection diagnosed with locally advanced oesophageal adenocarcinoma with patients with metastases showed that patients with metastasized disease had higher pN and pT stages. Interestingly, the specimens of patients with metastases showed significantly more chemoresistance (84%) compared with the specimens of patients without metastases (54%). These worse pathological findings resulted in a median overall survival of only 9 months in the patient cohort with metastases^16^. The median survival after resection with or without other therapy options of metastatic disease of oesophageal cancer in other retrospective non-comparative studies ranges between 9 and 22 months^17–22^. In the present patient cohort, patients who underwent resection either with or without other therapy had a median survival of 19.0 months. The surgically treated patients were associated with a significantly longer median overall survival compared with non-surgically treated patients (19.0 months *versus* 11.0 months respectively) (*P* < 0.001). To date, only a few comparative studies exist that confirm the present findings^23,24^. Ichida *et al*.^25^ even describe a median survival of 48 months after resection of metachronous pulmonary metastases compared with 10 months without resection. In multivariable Cox regression, the known risk factor pN status proved to be a factor for unfavourable survival in the present study. Furthermore, resection of metastases proved to be an independent factor for a more favourable outcome.

The present study also has limitations, such as its retrospective and single-centre design. Different starting points for the measurement of overall survival were used. The overall survival of patients who received best supportive care was measured from the time of diagnosis of the metastatic disease, in contrast to the beginning of therapy for all other patients. This could result in lead time bias, favouring patients who received best supportive care. However, these patients showed the worst survival in all of the analyses that were performed. Furthermore, due to the personalized therapy regimens, and the advent of targeted therapy options causing a significant change in systemic treatment in recent years^26^, it was not possible to further subdivide this patient cohort based on different systemic therapy options. Additionally, various radiotherapy regimens were included in the group of patients treated with radiotherapy, without subdividing into subgroups due to small numbers of patients. Moreover, only patients with metastatic disease who were diagnosed and treated at the University Hospital of Cologne were included in the present study, which suggests a potential selection bias. However, standardized follow-up schemes were implemented to counteract this bias. Additionally, the initial inclusion criterion of Ivor Lewis oesophagectomy implies a selection bias of initially fitter patients. This also applies to the selection of patients eligible for potential resection of metastases. To eliminate these limitations, further statistically sufficiently powered prospective studies are needed to prove the associations found in the present study. As a definition of oligometastatic disease did not exist at the beginning of the study interval, the present study did not include a currently used definition of oligometastatic disease^11,27^. Individualized treatment was conducted as local therapy for patients with limited metastatic disease, as defined above.

The data gathered show that long-term survival in stage IV oesophageal cancer after resection is possible. However, as both the present study and previously published studies^19,23,28^ have utilized heterogeneous study cohorts, it is crucial to establish selection criteria for eligible patients for local therapy. Subgroup analyses of the present study showed that patients with metachronous solid-organ metastases could benefit from multimodal therapy including resection. Even though the statistical power is limited due to small sample sizes, the present study involves one of the biggest patient cohorts regarding the treatment of metastatic oesophageal cancer.

Additionally, an independent patient cohort with PET-positive distant lymph node metastases was investigated as PET positivity can predict stage and prognosis^29^. The majority of these patients received neoadjuvant therapy, followed by resection. Interestingly, patients with histologically tumour-free lymph nodes had significantly better survival, regardless of their initial PET-positive lymph nodes. This could indicate that PET-positive distant lymph node metastases should not exclude patients from multimodal therapies with curative intent.

In conclusion, the present analysis suggests individualized treatments including surgery for patients with limited solid-organ metastases of oesophageal cancer as potential treatment options. However, prospective randomized studies, such as the RENAISSANCE trial^30^ that is currently recruiting, are needed to evaluate the importance of surgery in the treatment of stage IV oesophageal cancer.

## Supplementary Material

zrae054_Supplementary_Data

## Data Availability

The data sets generated and analysed during the present study are available from the corresponding author on reasonable request.
