# Supplementary material for: Surgical and multimodal treatment of metastatic oesophageal cancer: retrospective cohort study
Source: BJS Open. 2024 May 30;8(3):zrae054. doi: 10.1093/bjsopen/zrae054 (PMC11138957; doi:10.1093/bjsopen/zrae054)
Supplement: zrae054_Supplementary_Data [file zrae054_supplementary_data.docx]

**Surgical and multimodal treatment of metastatic oesophageal cancer: retrospective cohort study**

Karl Knipper^1^, Julian Lemties^1^, Thaddaeus Krey^1^, Su Ir Lyu^2^, Naita M. Wirsik^1^, Lars M. Schiffmann^1^, Hans F. Fuchs^1^, Florian Gebauer^1^, Wolfgang Schröder^1^, Felix C. Popp^1^, Alexander Quaas^2^, Hans A. Schlößer^1,3^, Christiane J. Bruns^1^, and Thomas Schmidt^1,*^

*^1^ Faculty of Medicine and University Hospital of Cologne, Department of General, Visceral and Cancer Surgery, University of Cologne, Cologne, Germany*

*^2^ Faculty of Medicine and University Hospital of Cologne, Institute of Pathology, University of Cologne, Cologne, Germany*

*^3^ Faculty of Medicine and University Hospital of Cologne, Center for Molecular Medicine Cologne, University of Cologne, Cologne, Germany*

**Corresponding author.**

Prof. Dr. Dr. Thomas Schmidt

thomas.schmidt1@uk-koeln.de

Uniklinik Köln

Allgemein-, Viszeral-, Tumor- und Transplantationschirurgie

Kerpener Straße 62

50937 Köln

Germany

**Supplementary Materials - Index**

| **Supplementary Figures and Tables** | |  | |
| --- | --- | --- | --- |
| Figure S1 | | *page 2* | |
| Figure S2 | | *page 3* | |
| Figure S3 | | *page 4* | |
| Table S1 | | *page 5* | |
| Table S2 | | *page 6* | |
| Table S3 | | *page 7* |  |
| Table S4 | | *page 8* | |
| Table S5 | | *page 9* | |

**Supplementary Figures and Tables**

**Figure S1**

Kaplan-Meier curves for overall survival depending on the intention of treatment in patients with (A) adenocarcinoma, (B) squamous cell carcinoma, (C) metachronous metastatic disease, (D) metachronous metastatic disease within one year after primary tumor resection, (E) metachronous metastatic disease after one year after primary tumor resection, and (F) synchronous metastatic disease.


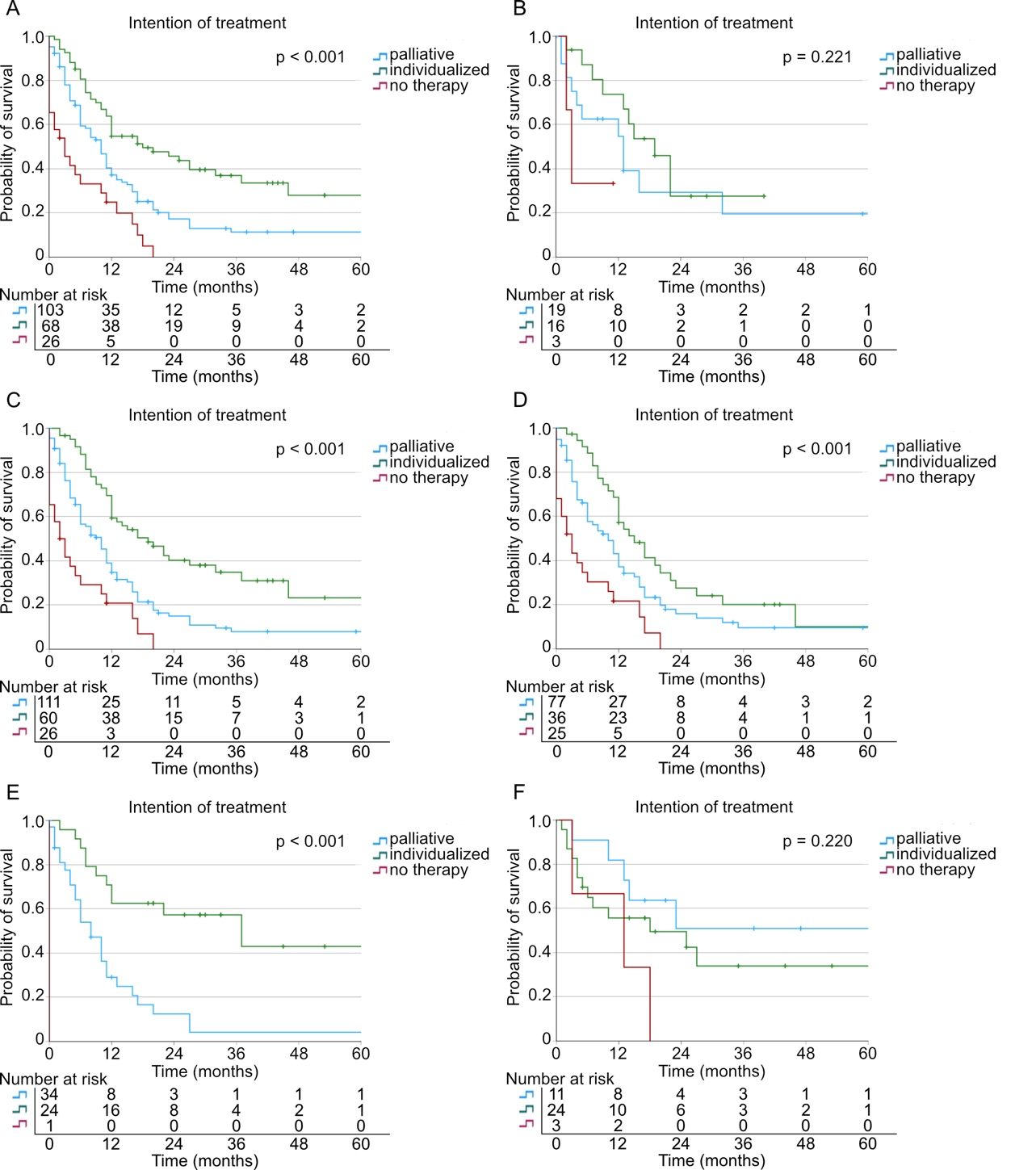


**Figure S2**

Kaplan-Meier curves for overall survival depending on the intention of treatment in patients with (A) liver metastases, (B) lung metastases, and (C) adrenal gland metastases.


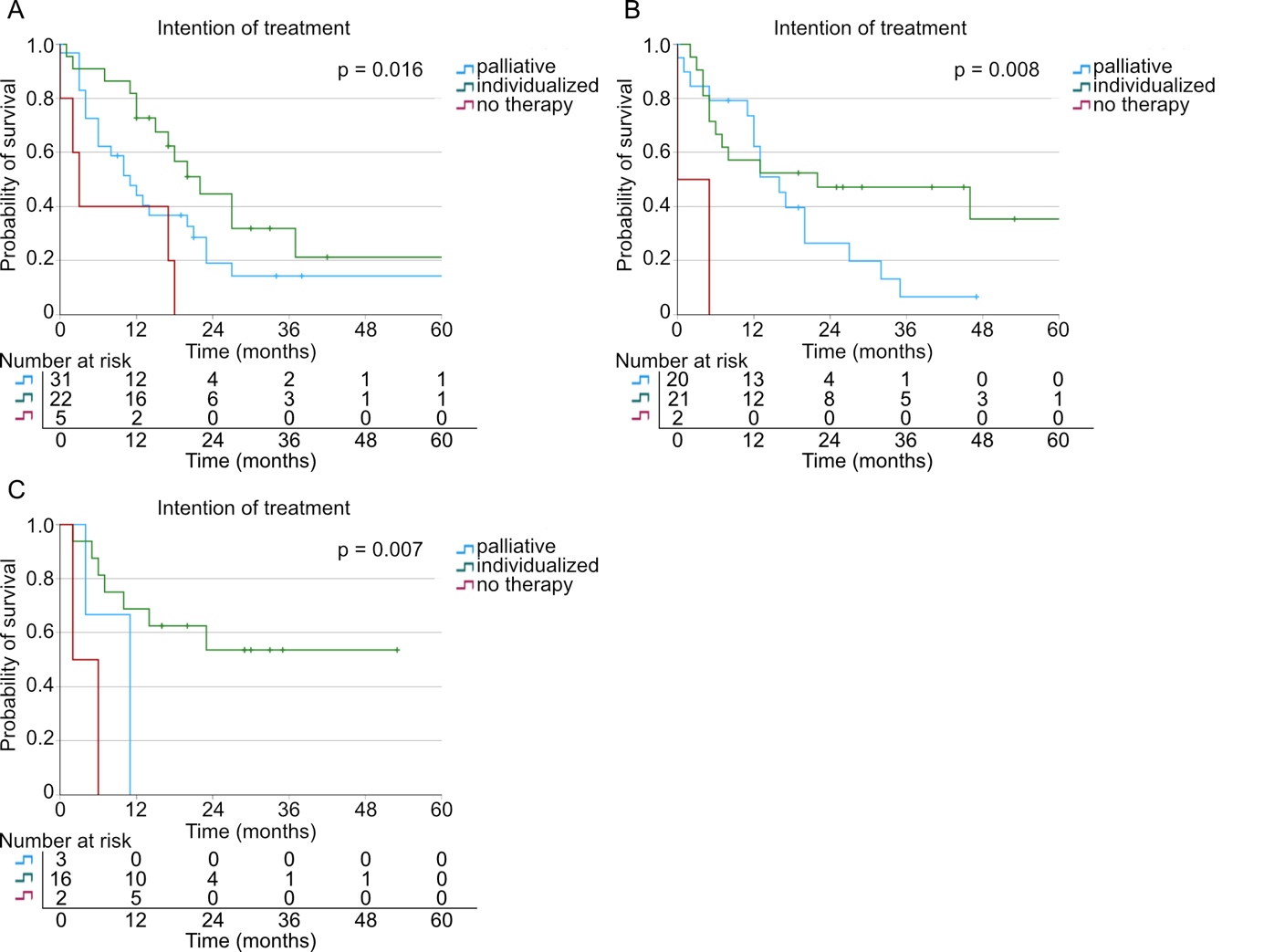


**Figure S3**

(A) Kaplan-Meier curve and (B) swimmer plot for overall survival of the included patient cohort with initially PET-positive lymph nodes. Cross: dead, arrow: alive.


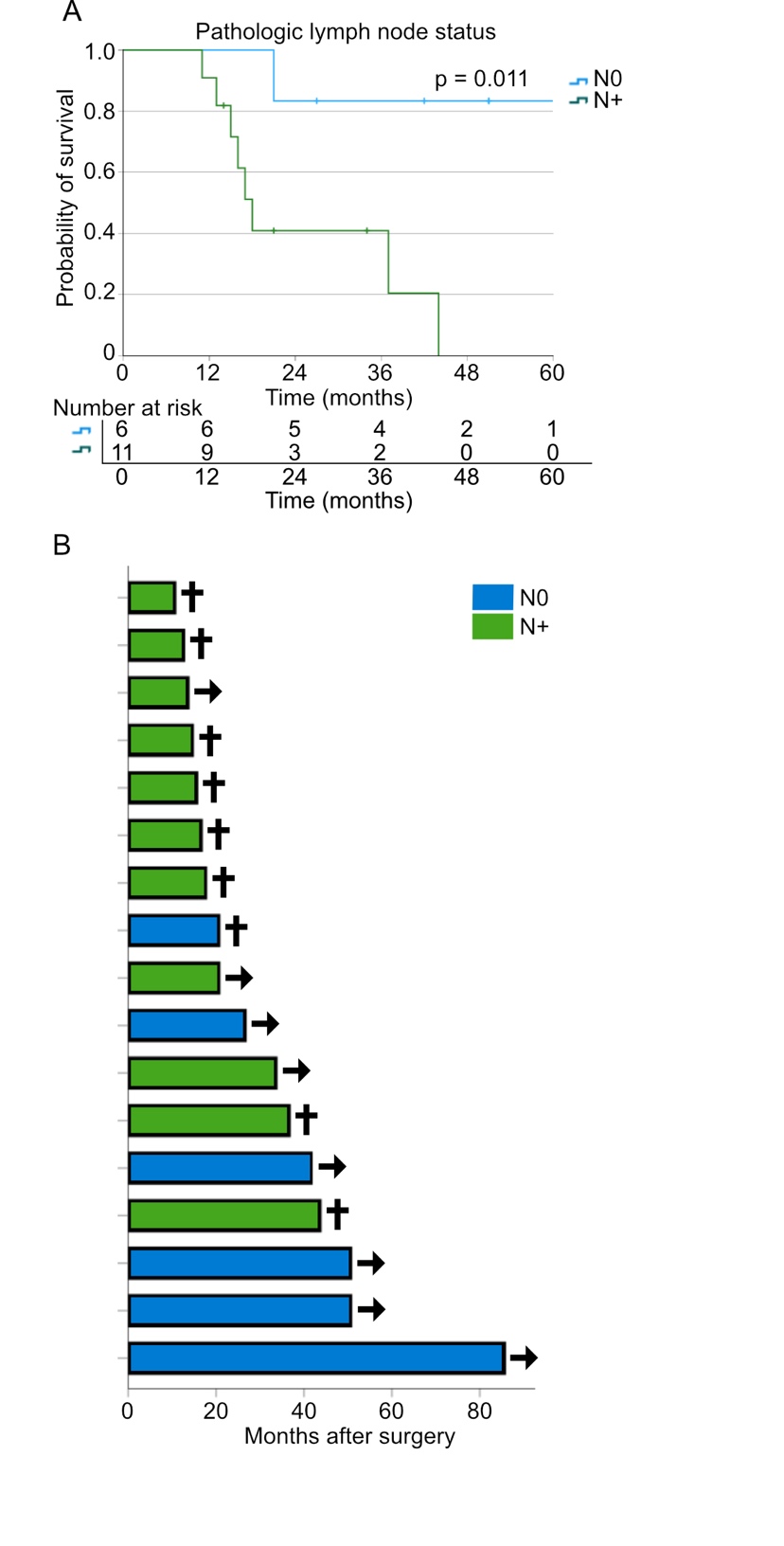


**Table S1**

A detailed listing of postoperative complications following Ivor-Lewis-esophagectomy of the total study population and patients with synchronous metastases divided into simultaneous metastases resection and no resection. ARDS: Acute respiratory distress syndrome. Values are n (%) unless otherwise indicated.

| **Characteristic** | **Total** | **Synchronous metastases** | |  |
| --- | --- | --- | --- | --- |
|  |  | **No resection** | **Resection** |  |
|  | **n (%)** | **n (%)** | **n (%)** | **p-value** |
| **No. of patients** | 235 (100.0) | 11 (100.0) | 24 (100.0) |  |
| **Anastomotic leakage** |  |  |  | 0.856 |
| No | 213 (90.6) | 9 (81.8) | 19 (79.2) |  |
| Yes | 22 (9.4) | 2 (18.2) | 5 (20.8) |  |
| **ARDS** |  |  |  | - |
| No | 233 (99.1) | 11 (100.0) | 24 (100.0) |  |
| Yes | 2 (0.9) | 0 (0.0) | 0 (0.0) |  |
| **Chylothorax** |  |  |  | - |
| No | 234 (99.6) | 11 (100.0) | 24 (100.0) |  |
| Yes | 1 (0.4) | 0 (0.0) | 0 (0.0) |  |
| **Enterothorax** |  |  |  | - |
| No | 231 (98.3) | 11 (100.0) | 24 (100.0) |  |
| Yes | 4 (1.7) | 0 (0.0) | 0 (0.0) |  |
| **Pylorospasm** |  |  |  | 0.070 |
| No | 174 (74.0) | 6 (54.5) | 20 (83.3) |  |
| Yes | 61 (26.0) | 5 (45.5) | 4 (16.7) |  |

**Table S2**

Metastatic site of the total cohort as well as the patient cohort with individualized or non-individualized intention of treatment of metastasis. Bold print marks p-value below 0.05. Values are n (%) unless otherwise indicated.

| **Characteristic** | **Total** | **Individualized** | **Non-individualized** |  |
| --- | --- | --- | --- | --- |
|  |  | **treatment** | **treatments** |  |
|  | **n (%)** | **n (%)** | **n (%)** | **p-value** |
| **No. of patients** | 235 (100.0) | 84 (100.0) | 151 (100.0) |  |
| **Metastatic site** |  |  |  | **<0.001** |
| Adrenal gland | 21 (8.9) | 16 (19.0) | 5 (3.3) |  |
| Bone | 24 (10.2) | 3 (3.6) | 21 (13.9) |  |
| Brain | 21 (8.9) | 12 (14.3) | 9 (5.9) |  |
| Kidney | 1 (0.4) | 1 (1.2) | 0 (0.0) |  |
| Liver | 58 (24.7) | 22 (26.2) | 36 (23.8) |  |
| Lung | 43 (18.3) | 21 (25.0) | 22 (14.6) |  |
| Lymph node (distant) | 25 (10.6) | 9 (10.7) | 16 (10.6) |  |
| Peritoneum | 25 (10.6) | 0 (0.0) | 25 (16.6) |  |
| Pleura | 11 (4.7) | 0 (0.0) | 11 (7.3) |  |
| Soft tissue | 5 (2.1) | 0 (0.0) | 5 (3.3) |  |
| Urine bladder | 1 (0.4) | 0 (0.0) | 1 (0.7) |  |

**Table S3**

A detailed listing of the administered systemic therapy regimes for the total patient cohort as well as patients with synchronous or metachronous metastatic disease. FLO: Fluorouracil, Leucovorin, Oxaliplatin, FLOT: Fluorouracil, Leucovorin, Oxaliplatin, Docetaxel, FOLFIRI: Fluorouracil, Leucovorin, Irinotecan, FOLFOX: Fluorouracil, Leucovorin, Oxaliplatin. Values are n (%) unless otherwise indicated.

| **Systemic therapy** | **Total** | **Synchronous** | **Metachronous** |
| --- | --- | --- | --- |
|  | **n (%)** | **n (%)** | **n (%)** |
| **No. of patients** | 127 (100.0) | 17 (100.0) | 110 (100.0) |
| Capecitabine, Oxaliplatin | 6 (4.7) | 0 (0.0) | 6 (5.5) |
| Fluorouracil, Cisplatin ± Docetaxel | 8 (6.3) | 0 (0.0) | 8 (7.3) |
| FLO | 8 (6.3) | 0 (0.0) | 8 (7.3) |
| FLOT | 46 (36.2) | 13 (76.5) | 33 (30.0) |
| FOLFIRI | 12 (9.5) | 0 (0.0) | 12 (10.9) |
| FOLFOX | 7 (5.5) | 1 (5.9) | 6 (5.5) |
| Nivolumab | 5 (3.9) | 0 (0.0) | 5 (4.5) |
| Paclitaxel, Carboplatin | 7 (5.5) | 0 (0.0) | 7 (6.4) |
| Pembrolizumab | 2 (1.6) | 0 (0.0) | 2 (1.8) |
| Ramucirumab ± Paclitaxel | 4 (3.2) | 0 (0.0) | 4 (3.6) |
| Trastuzumab mono | 3 (2.4) | 0 (0.0) | 3 (2.7) |
| Trastuzumab + chemotherapy | 7 (5.5) | 2 (11.8) | 5 (4.5) |
| Others (chemotherapy only) | 13 (10.3) | 1 (5.9) | 12 (10.9) |

**Table S4**

Univariable Cox regression. AC: adenocarcinoma, AEG: sievert classification of the adenocarcinoma of the esophagogastric junction, ASA: Grading of patients for surgical procedures of the American Society of Anesthesiologists, SCC: squamous cell carcinoma. Bold print marks p-value below 0.05.

| **Characteristic** | **Borders** | **Hazard Ratio** | **95 % confidence interval** | **p - value** |
| --- | --- | --- | --- | --- |
| **Gender** | female vs male | 1.079 | 0.715 - 1.630 | 0.717 |
|  |  |  |  |  |
| **Age (years)** | ≥ 65 vs < 65 | 1.232 | 0.898 - 1.689 | 0.196 |
|  |  |  |  |  |
| **ASA** | ≥ 2 vs 1 | 1.092 | 0.835 - 1.428 | 0.520 |
|  |  |  |  |  |
| **Histology** | SCC vs AC | 0.817 | 0.526 - 1.269 | 0.368 |
|  |  |  |  |  |
| **AEG** | ≥ 2 vs 1 | 0.975 | 0.688 - 1.381 | 0.887 |
|  |  |  |  |  |
| **Neoadjuvant therapy** | yes vs no | 0.508 | 0.318 - 0.814 | **0.005** |
|  |  |  |  |  |
| **pT** | ≥ 2 vs 1 | 1.166 | 1.016 - 1.337 | **0.029** |
|  |  |  |  |  |
| **pN** | ≥ 1 vs 0 | 1.274 | 1.112 - 1.461 | **<0.001** |
|  |  |  |  |  |
| **R** | ≥ 1 vs 0 | 3.306 | 1.615 - 6.771 | **0.001** |
|  |  |  |  |  |
| **Lymphatic vessel invasion** | 1 vs 0 | 1.438 | 1.041 - 1.986 | **0.028** |
|  |  |  |  |  |
| **Blood vessel invasion** | 1 vs 0 | 1.362 | 0.881 - 2.107 | 0.165 |
|  |  |  |  |  |
| **Metachronous metastases** | no vs yes | 0.547 | 0.346 - 0.864 | **0.010** |
|  |  |  |  |  |
| **Time of metachronous metastases (months)** | ≥ 13 vs 13 | 0.721 | 0.500 - 1.040 | 0.073 |
|  |  |  |  |  |
| **Number of metastases** | ≥ 2 vs 1 | 1.470 | 1.152 - 1.875 | **0.002** |
|  |  |  |  |  |
| **Number of metastatic sites** | ≥ 2 vs 1 | 1.419 | 1.120 - 1.798 | **0.004** |
|  |  |  |  |  |
| **Resection** | resection vs no resection | 0.544 | 0.384 - 0.772 | **<0.001** |

**Table S5**

General patient characteristics and clinicopathological characteristics of the total study population with PET-positive lymph nodes as well as the divided cohort into patients without or with pathologically diagnosed lymph node metastases. Adj.: adjuvant, AEG: sievert classification of the adenocarcinoma of the esophagogastric junction, ASA: Grading of patients for surgical procedures of the American Society of Anesthesiologists, LAD: Lymphadenectomy, LN: lymph node, neo.: neoadjuvant. Bold print marks p-value below 0.05. Values are n (%) unless otherwise indicated.

| **Characteristic** | **Total** | **pN0** | **pN+** |  |
| --- | --- | --- | --- | --- |
|  | **n (%)** | **n (%)** | **n (%)** | **p-value** |
| **No. of patients** | 17 (100.0) | 6 (100.0) | 11 (100.0) |  |
| **Sex** |  |  |  | 0.938 |
| Male | 14 (84.2) | 5 (83.3) | 9 (81.8) |  |
| Female | 3 (17.6) | 1 (16.7) | 2 (18.2) |  |
| **Age** |  |  |  | 0.394 |
| < 65 | 12 (70.6) | 5 (83.3) | 7 (63.6) |  |
| ≥ 65 | 5 (29.4) | 1 (16.7) | 4 (36.4) |  |
| **ASA** |  |  |  | 0.643 |
| 1 | 1 (5.9) | 0 (0.0) | 1 (9.1) |  |
| 2 | 9 (52.9) | 3 (50.0) | 6 (54.5) |  |
| 3 | 6(35.3) | 3 (50.0) | 3 (27.3) |  |
| 4 | 1 (5.9) | 0 (0.0) | 1 (9.1) |  |
| **Histology** |  |  |  | 0.622 |
| Adenocarcinoma | 13 (76.5) | 5 (83.3) | 8 (72.7) |  |
| Squamous cell carcinoma | 4 (23.5) | 1 (16.7) | 3 (27.3) |  |
| **AEG** |  |  |  | 0.506 |
| 1 | 8 (47.1) | 3 (50.0) | 5 (45.5) |  |
| 2 | 4 (23.5) | 1 (16.7) | 3 (27.3) |  |
| 3 | 1 (5.9) | 1 (16.7) | 0 (0.0) |  |
| Unknown/not applicable | 4 (23.5) | 1 (16.7) | 3 (27.3) |  |
| **Neoadjuvant therapy** |  |  |  | 0.159 |
| No | 3 (17.6) | 0 (0.0) | 3 (27.3) |  |
| Yes | 14 (82.4) | 6 (100.0) | 8 (72.7) |  |
| **pT** |  |  |  | 0.083 |
| 0 | 2 (11.8) | 2 (33.3) | 0 (0.0) |  |
| 1 | 6 (35.3) | 3 (50.0) | 3 (27.3) |  |
| 2 | 2 (11.8) | 0 (29.5) | 2 (18.2) |  |
| 3 | 7 (41.2) | 1 (16.7) | 6 (54.5) |  |
| **pN** |  |  |  | - |
| 0 | 6 (35.3) | 6 (100.0) | 0 (0.0) |  |
| 1 | 5 (29.4) | 0 (0.0) | 5 (45.5) |  |
| 2 | 4 (23.5) | 0 (0.0) | 4 (36.4) |  |
| 3 | 2 (11.8) | 0 (0.0) | 2 (18.2) |  |
| **R** |  |  |  | 0.539 |
| 0 | 15 (88.2) | 6 (100.0) | 9 (81.8) |  |
| 1 | 1 (5.9) | 0 (0.0) | 1 (9.1) |  |
| 2 | 1 (5.9) | 0 (0.0) | 1 (9.1) |  |
| **L** |  |  |  | **0.011** |
| 0 | 10 (58.8) | 6 (100.0) | 4 (36.4) |  |
| 1 | 7 (41.2) | 0 (0.0) | 7 (63.6) |  |
| **V** |  |  |  | **0.049** |
| 0 | 12 (70.6) | 6 (100.0) | 6 (54.5) |  |
| 1 | 5 (29.4) | 0 (0.0) | 5 (45.5) |  |
| **Localization of positive LN** |  |  |  | 0.116 |
| Cervical | 5 (29.4) | 3 (50.0) | 2 (18.2) |  |
| Retroclavicular | 5 (29.4) | 0 (0.0) | 5 (45.5) |  |
| Paraaortal | 6 (35.3) | 2 (33.3) | 4 (36.4) |  |
| Retroclavicular + paraaortal | 1 (5.9) | 1 (16.7) | 0 (0.0) |  |
| **Treatment of positive LN** |  |  |  | 0.606 |
| Neo. Radiochemotherapy | 1 (5.9) | 1 (16.7) | 0 (0.0) |  |
| Extended LAD | 6 (35.3) | 2 (33.3) | 4 (36.4) |  |
| Adj. radiotherapy | 7 (41.2) | 2 (33.3) | 5 (45.5) |  |
| Adj. chemotherapy | 2 (11.8) | 1 (16.7) | 1 (16.7) |  |
| Active surveillance | 1 (5.9) | 0 (0.0) | 1 (9.1) |  |
| **Pathological outcome extended LAD** |  |  |  | - |
| Negative | 3 (100.0) | 3 (100.0) | 0 (0.0) |  |
| Positive | 3 (100.0) | 0 (0.0) | 3 (100.0) |  |
